# Intraoperative 3D imaging leads to substantial revision rate in management of tibial plateau fractures in 559 cases

**DOI:** 10.1186/s13018-019-1286-7

**Published:** 2019-07-24

**Authors:** Nils Beisemann, Holger Keil, Benedict Swartman, Marc Schnetzke, Jochen Franke, Paul Alfred Grützner, Sven Y. Vetter

**Affiliations:** MINTOS—Medical Imaging and Navigation in Trauma and Orthopaedic Surgery, BG Trauma Center Ludwigshafen at Heidelberg University Hospital, Ludwig-Guttmannstr. 13, 67071 Ludwigshafen, Germany

**Keywords:** Tibial plateau fracture, Cone beam CT, Intraoperative imaging, 3D scan

## Abstract

**Introduction:**

The aim of this study was to evaluate the intraoperative revision rate and reasons for revision following 3D imaging in the management of dislocated articular tibial plateau fractures based on a large patient sample.

**Methods:**

This retrospective cohort study included all patients who underwent open reduction and internal fixation due type B or C tibial plateau fracture according to the AO/OTA classification between August 2001 and December 2017 using intraoperative cone beam CT (3D imaging) for the analysis of fracture reduction and implant placement.

The findings of the 3D scan were categorized regarding the amount and type of revision. Furthermore, demographic data was examined.

**Results:**

Five hundred and fifty-nine consecutive fractures were included in the study. Evaluation of the image data records revealed an intraoperative revision due to the usage of 3D imaging in 148 out of 559 cases (26.5%). The most common reasons for an intraoperative revision were insufficient fracture reduction (114 cases) and screw length (21 cases).

**Conclusion:**

This study reveals indications for a limited analysis of fracture reduction and implant placement during the operative treatment of dislocated articular tibial plateau fractures using conventional fluoroscopy. In view of the high revision rate during open reduction and internal fixation of tibial plateau fractures due to 3D imaging the usage of intraoperative cone beam, CT may be considered. If this is not possible, a postoperative computed tomography may therefore be reasonable.

## Introduction

Tibial plateau fractures account for around 1% of all fractures and can be treated conservatively or surgically, depending on the fracture morphology [1, 2]. For dislocated articular tibial plateau fractures, surgical management is regarded as the gold standard.

Various osteosynthesis procedures and implants are available for the surgical management. For the intraoperative analysis of fracture reduction and implant placement, conventional fluoroscopy is generally applied [3]. However, the standard fluoroscopy has limitations with regard to displaying the complex anatomy of the tibial head. Due to the concave shape of the tibial plateau, parts of the tibial head are often overlaid by other bony structures in the beam path of two-dimensional images (this is known as the “summation effect”). As a result, a sufficient assessment of the complete articular surface may not be possible [4].

Postoperative computed tomography (CT) is therefore recommended to evaluate the surgical result [5]. If inadequate implant positioning or insufficient reduction is observed in these images a revision surgery may be necessary. Intraoperative 3D imaging provides additional information and displays sectional images similar to computed tomography detecting malreduction and implant malposition that were not visible in conventional fluoroscopy [6, 7].

Until now, only a few studies have been published on intraoperative 3D imaging in the treatment of proximal tibia fractures. The benefits of 3D imaging have been demonstrated in various anatomical regions based on a large cohort. 3D imaging led to a revision rate of 32.7% in syndesmotic lesions and up to 40.3% in calcaneal fractures [8, 9]. With regard to the consequences of using intraoperative 3D imaging techniques in tibial plateau fractures, very few studies exist. Furthermore, relatively small numbers of fractures were investigated; in some studies, proximal tibia fracture was only analyzed as a subgroup. These publications report a revision rate from 11 to 21% based on 3D imaging [10–13].

The aim of this study was to evaluate the intraoperative revision rate and reasons for revision following 3D imaging in the management of displaced articular tibial plateau fractures based on a large cohort. The hypothesis was that insufficient reduction or implant malposition may not be visible in conventional fluoroscopy but can be visualized in intraoperative 3D imaging.

## Material and methods

The retrospective study included all patients who underwent surgery for type B or C tibial plateau fracture according to the AO/OTA classification between August 2001 and December 2017 and whose results were verified using intraoperative 3D imaging.

The patients were positioned supine on a radiolucent carbon-fiber table, and a tourniquet was applied. Depending on the fracture type, an anterolateral, anteromedial, central, or posteromedial surgical approach was performed for exposure. After open reduction, an internal plate fixation was carried out.

**Fig. 1:**
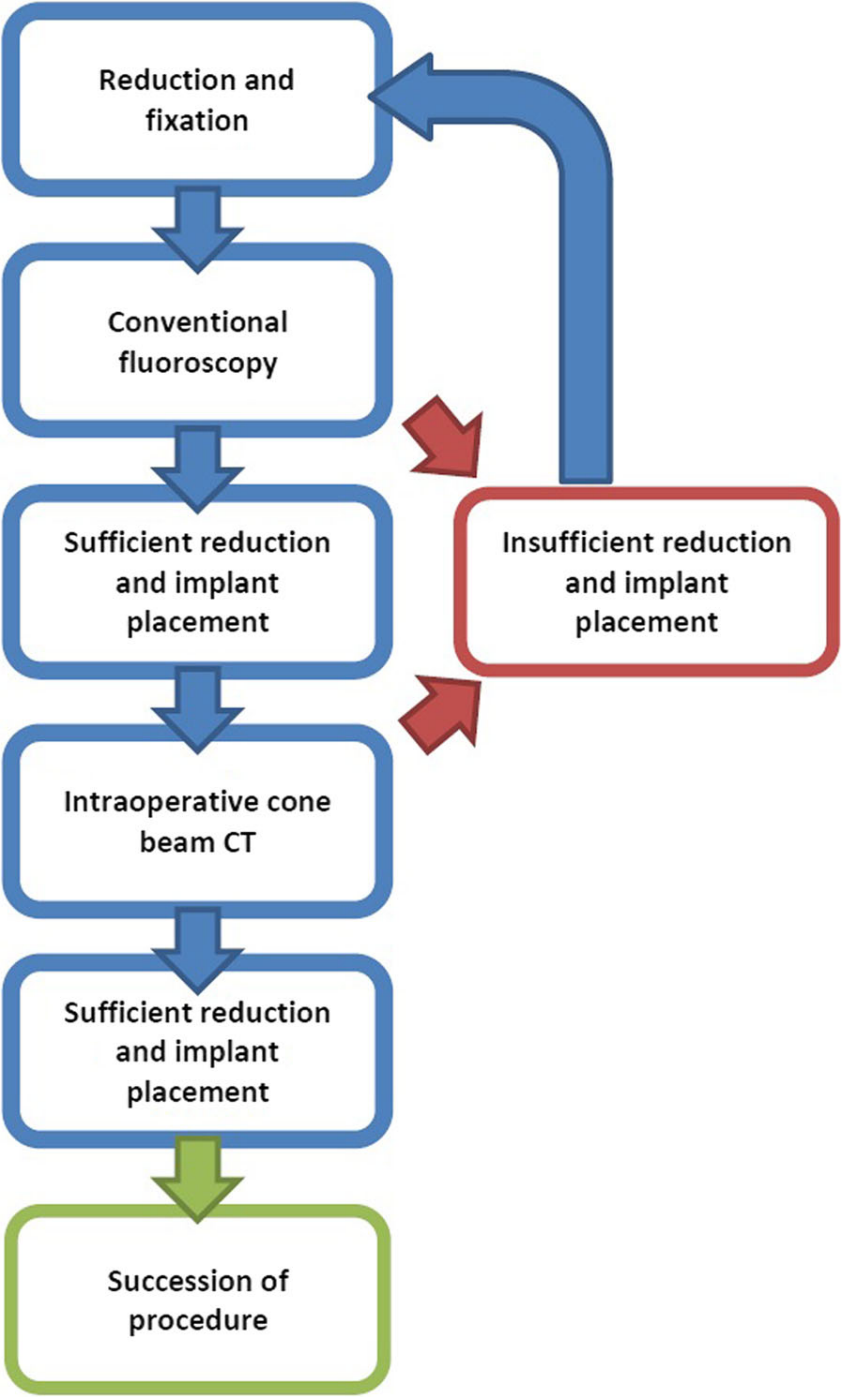
Workflow for the intraoperative 3D scan

The control of the reduction was then performed, initially with 2D fluoroscopy. If the surgeon was satisfied with the reduction result and implant placement in conventional fluoroscopy, a 3D scan was performed with a cone beam CT (Fig. 1).

Initially, a SIREMOBILE Iso-C 3D (Siemens, Erlangen, Germany) was used for this purpose. From 2005 onwards, an ARCADIS Orbic 3D (Siemens, Erlangen, Germany) was utilized.

**Fig. 2:**
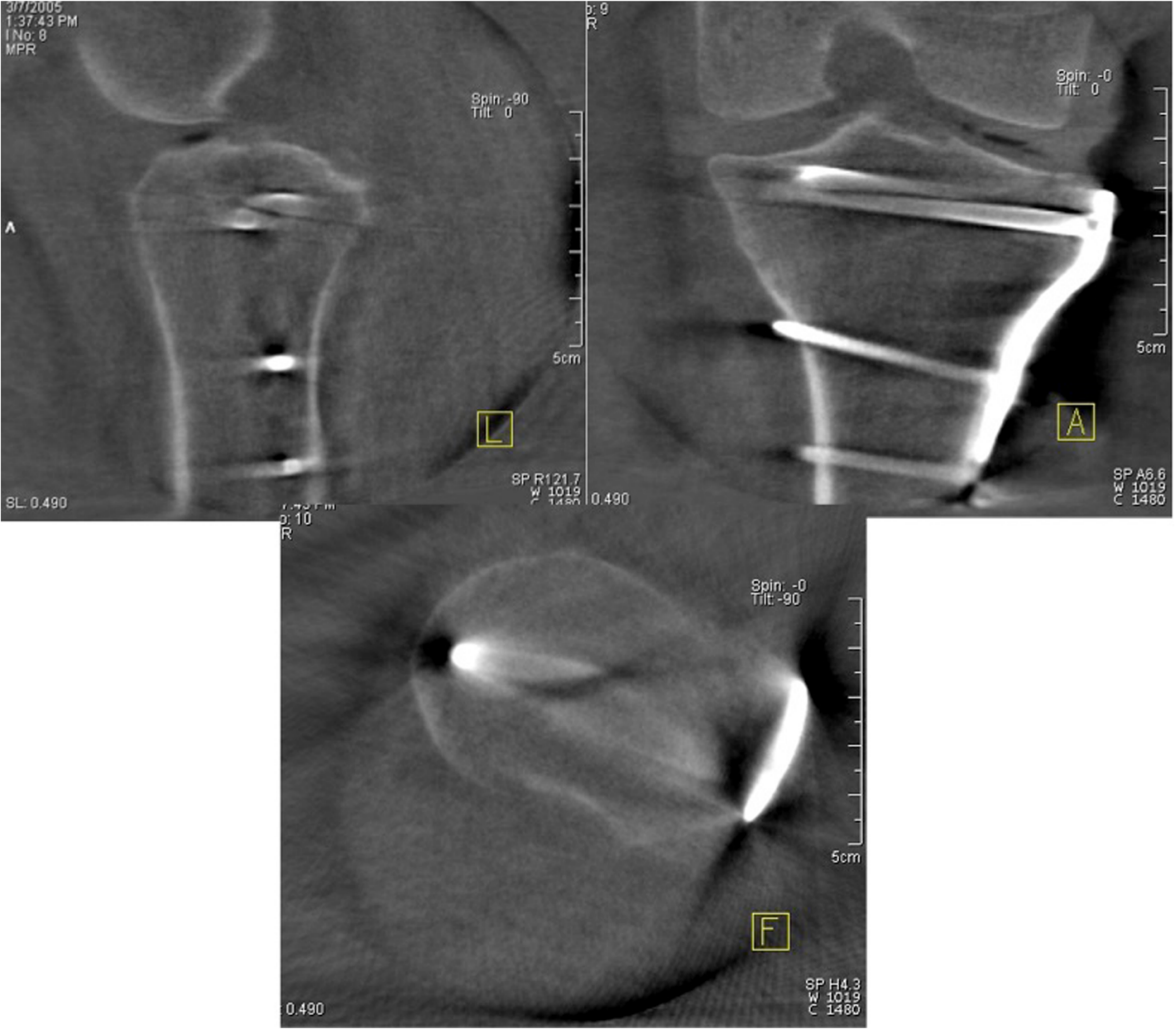
3D scan of the tibial plateau. For analysis, the sagittal, transverse and frontal planes were set, based on anatomical landmarks in order to obtain an orientation over the joint

The two 3D C-arms share the same operating principle:

The motorized C-arm performs a 190° orbital movement and produces 100 two-dimensional images. These images are then combined in a 3D dataset and processed into sectional images. Within the 3D dataset, any desired plane may be set. In practice, the sagittal, transverse, and frontal planes are adjusted initially based on anatomical landmarks (Fig. 2).

Similar to a CT scan, it is possible to analyze the planes layer by layer and thus gain an overview of the reduction results and the implant position.

The scan time lasts for 1 min (ARCADIS Orbic 3D) or 2 min (SIREMOBILE Iso-C 3D). Including the evaluation of the images and decision making, the total additional intraoperative time approximates to 5 min. To view specific anatomical regions, the pre-set “standard plane” can be exited and realigned as required. Step-offs and splits can also be measured via the screen.

**Fig. 3:**
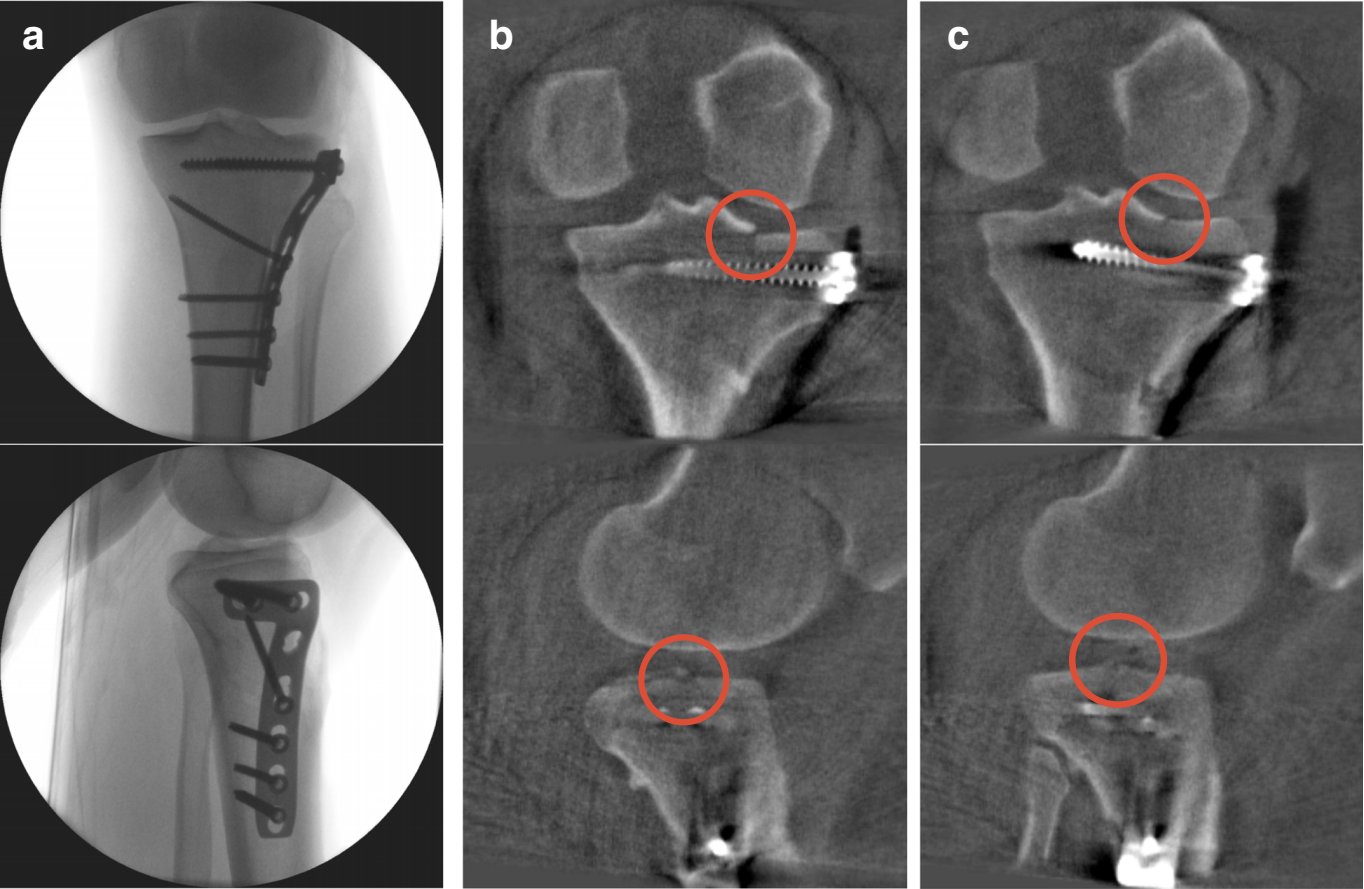
**a** Intraoperative fluoroscopic images in a.p. and lateral view, with apparently stepless reduction. **b** Subsequent 3D scan with significant step in the joint. **c** 3D scan after revision, now stepless reduction

If a malpositioned implant or insufficient reduction was observed during the assessment of the scan, a correction was performed immediately. Again, the reduction and implant position was evaluated using conventional fluoroscopy in the two standard planes. If the assessment did not reveal a malalignment or malposition of the implant in conventional fluoroscopy, a 3D scan was performed again (Figs. 1 and 3).

The result of each scan and the ensuing consequences were documented in the immediate postoperative period by the surgeon while still in the operating room. The revisions were classified into four categories:

1. Improvement in reduction with articular step-offs of > 2 mm
2. Replacement of an intraarticular screw
3. Replacement of a screw with one of a different length. Here, either a shorter screw was inserted to correct a projection of more than 4 mm on the opposite side or a longer screw was used to correct defective fixation of a fragment.
4. “Other consequence”

In addition to the database analysis, demographic data and the side affected by the fracture and were also studied. The evaluation was conducted using the Excel spreadsheet software (Microsoft, Redmond, USA).

## Results

Five hundred and fifty-nine consecutive fractures were included in the study. Most of these were type B3 (37.4%) and C3 (30.4%) fractures. These were followed by type B2 (15.9%) and B1 (7.9%) fractures. The least common were type C2 (4.5%) and C1 (4.3%) fractures. Two hundred and twelve fractures (37.9%) affected the right side and 347 fractures (62.1%) the left side.

The demographic data is detailed in Table 1.

**Table 1:** Demographic data

Age [years (range; SD)]	48.1 (14–85; 14.57)
Female [*n* (%)]	224 (40.1%)
Male [*n* (%)]	335 (59.9%)
BMI [kg/m^2^ (range; SD)]	26.4 (18–49; 4.87)

The evaluation of the data revealed in 148 out of 559 cases an immediate intraoperative revision after using intraoperative 3D imaging. The revision rate accounted for 26.5% (157 revisions in total). An improved reduction result was achieved in 114 cases. An intraarticular screw was replaced in five cases. A different screw length was used in 21 cases: in 17 of these cases, a shorter screw was inserted because the original one had projected by more than 4 mm on the opposite side, and in four cases, the original screw did not fix the targeted fragment fully so a longer screw was chosen. The category “other consequences” was broken down as follows:

- In three cases, an intraarticular bone fragment was removed which was not visible in conventional fluoroscopy.
- In two further cases, an additional screw was inserted to support the reduction result.
- In one case, a plate projected proximally and was corrected,
- In five cases, the position of a screw was altered to achieve better fixation of a fragment.
- In two cases, an additional defect was discovered which was not visible in conventional fluoroscopy.
- In one case, a lateral fragment could not be fixed and had to be discarded.
- In one case, it was found that a central fragment could not be reached via the lateral approach making an additional dorsal approach necessary.
- In one case, it turned out that an additional plate was necessary to achieve stabilization.

The distribution of intraoperative revisions divided by fracture types is shown in Table 2.

**Table 2:** Intraoperative revisions divided by fracture types

Fracture classification	B1	B2	B3	C1	C2	C3
Number of operations	44	89	207	24	25	170
Number with at least one intraoperative consequence	8	22	57	2	4	55
Proportion (in%) with at least one intraoperative consequence	18.2	24.7	27.5	8.3	16	32.4

## Discussion

The aim of our study was to determine the revision rate and reasons for revision following intraoperative 3D imaging in the management of tibial plateau fractures.

Our hypothesis was that insufficient reductions or implant malpositions which are not visible in conventional fluoroscopy may be visualized by intraoperative 3D imaging.

In our study, at least one revision was performed based on 3D imaging in 26.5% of the cases.

The largest number of tibial plateau fractures previously included in an intraoperative 3D imaging study was 32 and patient numbers in other studies were lower [10]. One reason for this was that tibial plateau fractures were viewed as a subgroup. Ruan et al. and Kendoff et al. obtained similar results for the revision rate (20% and 21%, respectively) as observed in this study [11, 13].

Conversely, studies by Kendoff et al. and Atesok et al. looking at the tibial plateau group showed lower revision rates of 12.5% and 11.7%, respectively [10, 12]. In these studies, the lower number of intraoperative consequences might be due to a different benchmark for the necessity of revision. Just like in our study, Kendoff et al. used an articular step-off of > 2 mm as a reason for a further correction. The other sources available to us contain no specific information in this regard.

The main reason for revision in our study was an improvement in reduction (72.6% of cases). This is confirmed by comparison with other studies, where the main reason for revision was also a correction of reduction [11]. These rates are comparable to our findings. However, in the two patients requiring revision in the study from Atesok et al. [12], an additional screw was inserted or the position and length of a screw were altered.

If the distribution of intraoperative consequences between individual fracture categories according to the AO/OTA classification is considered, it becomes clear that large numbers of revisions were performed, mainly in type C3 (32.4%) and B3 fractures (27.5%). This is because B3 and C3 fractures are split-depression and multifragmentary fractures, respectively, in which the tibial head is severely damaged and the articular surface is indented and/or fractured into multiple fragments. Due to the summation effects, conventional fluoroscopy is limited in these cases, as the complexity of the fracture significantly altered the articular surface. This problem has already been demonstrated in the proximal tibia and other anatomical regions on the basis of specimen studies [4, 14, 15]. Based on the representation of the surgical site in sectional planes and the free choice of viewing angle, 3D imaging therefore offers significant advantages in evaluating the reduction result. Remaining step-offs or splits, which were not visible in conventional fluoroscopy, can therefore be detected, and a correction can be achieved. Nevertheless, a benefit was also found when considering other fracture types. Except in the case of type C1 fractures, the revision rate was at least 16%. This additionally underlines the advantage of intraoperative 3D imaging over conventional fluoroscopy in the management of less complex fractures.

Another important finding was the detection of intraarticular bone fragments in three cases. Without 3D imaging, these fragments would have remained undetected in conventional fluoroscopy. This problem has not been described previously in the tibial plateau region, although Atesok et al. did report similar findings in the hip joint. In a Pipkin I fracture, a fragment between acetabulum and femoral head had remained undetected in conventional fluoroscopy [12].

There exists evidence that 3D imaging offers an advantage over conventional imaging for all dislocated articular tibial plateau fractures, as it improves the visualization of insufficient anatomical reduction, malpositioned implants, and intraarticular fragments. Since an immediate correction is possible, a subsequent surgery or an impaired clinical and radiological outcome can thus be prevented.

A comparison of the demographic data from this study with other studies revealed no anomalies with regard to age, weight, or gender distribution [2, 16].

A possible limitation of this study is the restricted C-arm image. Unlike computed tomography, it can only display a predefined section measuring 12 × 12 × 12 cm. This means, for example, that fracture splits extending into the tibial diaphysis cannot be assessed. However, because such splits occur in only a very few cases, the display size is usually sufficient to analyze tibial plateau fractures. Additionally, the limit of an articular step-off of > 2 mm—in which correction is performed—is debatable; the recommendations in the literature range from 1 to 4 mm [17, 18]. However, it has already been demonstrated in other anatomical regions that an articular step-off of more than 2 mm leads to an increased risk of arthritis [19, 20].

Furthermore, the two three-dimensional scanners that were used in this study differ in the time required for the scanning procedure. In addition, the ARCADIS Orbic 3D C-arm appears to provide improved image quality than the Iso-C 3D. However, in this study, we found that this did not influence the decision as to whether the alignment of the reconstructed tibia plateau was correct.

A further limitation of this study is the lack of proof of the clinical benefit. Only the radiological consequences resulting from the intraoperative 3D imaging were examined and not the clinical outcome.

Furthermore, it should be considered whether the benefit of intraoperative 3D imaging could have been better verified by case control analysis without a 3D scan in the control group. However, this was rejected for ethical reasons as a worse clinical outcome was assumed. It has to be emphasized though that an improved reduction and implant placement generally affects the clinical outcome positively.

In addition, the consequences of a prolonged operation time of about 5 min per scan have not been investigated.

## Conclusion

This study revealed that the correct alignment of the tibial plateau is difficult to evaluate using conventional fluoroscopy. In view of the high intraoperative revision rate, intraoperative 3D imaging appears to be beneficial for the analyzation of reduction and implant placement. If intraoperative 3D imaging is not available, a postoperative computed tomography should be considered.

## Data Availability

The datasets used and analyzed during the current study are available from the corresponding author on reasonable request.
